# Citrus By-Products as a Valuable Source of Biologically Active Compounds with Promising Pharmaceutical, Biological and Biomedical Potential

**DOI:** 10.3390/ph16081081

**Published:** 2023-07-29

**Authors:** Silvija Šafranko, Drago Šubarić, Igor Jerković, Stela Jokić

**Affiliations:** 1 Faculty of Food Technology Osijek, University of Osijek, Franje Kuhača 18, 31000 Osijek, Croatia; silvija.safranko@ptfos.hr (S.Š.); dsubaric@ptfos.hr (D.Š.); 2 Department of Organic Chemistry, Faculty of Chemistry and Technology, University of Split, Ruđera Boškovića 35, 21000 Split, Croatia

**Keywords:** citrus by-products, citrus anatomy, health benefits, bioactive compounds

## Abstract

Citrus fruits processing results in the generation of huge amounts of citrus by-products, mainly peels, pulp, membranes, and seeds. Although they represent a major concern from both economical and environmental aspects, it is very important to emphasize that these by-products contain a rich source of value-added bioactive compounds with a wide spectrum of applications in the food, cosmetic, and pharmaceutical industries. The primary aim of this review is to highlight the great potential of isolated phytochemicals and extracts of individual citrus by-products with bioactive properties (e.g., antitumor, antimicrobial, antiviral, antidiabetic, antioxidant, and other beneficial activities with health-promoting abilities) and their potential in pharmaceutical, biomedical, and biological applications. This review on citrus by-products contains the following parts: structural and chemical characteristics; the utilization of citrus by-products; bioactivities of the present waxes and carotenoids, essential oils, pectins, and phenolic compounds; and citrus by-product formulations with enhanced biocactivities. A summary of the recent developments in applying citrus by-products for the treatment of different diseases and the protection of human health is also provided, emphasizing innovative methods for bioaccessibility enhancements (e.g., extract/component encapsulation, synthesis of biomass-derived nanoparticles, nanocarriers, or biofilm preparation). Based on the representative phytochemical groups, an evaluation of the recent studies of the past six years (from 2018 to 2023) reporting specific biological and health-promoting activities of citrus-based by-products is also provided. Finally, this review discusses advanced and modern approaches in pharmaceutical/biological formulations and drug delivery (e.g., carbon precursors for the preparation of nanoparticles with promising antimicrobial activity, the production of fluorescent nanoparticles with potential application as antitumor agents, and in cellular imaging). The recent studies implementing nanotechnology in food science and biotechnology could bring about new insights into providing innovative solutions for new pharmaceutical and medical discoveries.

## 1. Introduction

Citrus fruits—more precisely, the genus *Citrus* L., which belongs to the subfamily Aurantioideae in the family Rutaceae—represent the major fruit crops commercially cultivated worldwide [1,2]. These fruits are widely known for their high health-benefiting properties, which is of great importance, since citrus fruits are the most widely consumed fruits globally [3,4,5,6]. The cultivation of *Citrus* genus includes the species such as lemon (*C. limon* (L.) Osbeck), sweet orange (*C. sinensis* (L.) Osbeck), mandarin (*C. reticulata* Blanco), grapefruit (*C. paradisi* Macfad.), pomelo (*C. maxima* (Burm.) Merr.), citron (*C. medica* L.), lime (*C. aurantiifolia* (Christm.) Swingle), and bergamot (*C. × bergamia* Risso & Poiteau) [4,5,7,8,9]. Although a significant part of the industrially processed citrus fruits is used to produce essential oils and juice, citrus-based candies, jellies, and extracts production also represent key factors for the food industry [5]. A large quantity of waste and by-products yearly produced during citrus processing has become a fundamental concern from both economical and environmental aspects. Citrus processing generates over 15 million tons of residue, mostly in the form of peels, seeds, and membranes [10,11]. Therefore, citrus waste valorization and proper industrial waste management are highly encouraged, being also a high-research priority topic for the scientific community recently. There have been many scientific reports related to the potential and beneficial utilization of industrial by-products, mainly dealing with innovative extraction methods for obtaining extracts rich in bioactive compounds [1,2,3,4,10,11,12]. These extracts have shown versatile health beneficial activities, due to the high content of biologically active compounds naturally present in the extracts [13,14]. Numerous studies have considered citrus extracts as a natural source of bioactive components exhibiting beneficial activities, including antioxidant [15,16,17,18,19], antibacterial [16,18,19,20,21], antidiabetic [17,22,23,24], neuroprotective [22,25,26,27], and anti-inflammatory [28,29,30,31] activities, as well as antitumor [32,33,34,35] potential. Also, citrus by-products are considered a valuable source of phytochemicals (such as *d*-limonene, essential oils, phenolic acids, carotenoids, vitamins, minerals, and flavonoids), which, isolated or in the form of mixtures/extracts, could exhibit versatile biological activities especially beneficial for the food industry [36,37,38]. Citrus-based essential oils exhibit significant antimicrobial activity against foodborne bacteria and also antioxidant activity to prevent the effects of oxidation; hence, citrus-based essential oils could act as natural preservatives [39,40,41]. Furthermore, several studies have reported the high repellent activity [42,43,44] and fumigant toxicity [45,46] of citrus-based essential oils against different insects.

It is well known that the presence of phenolic compounds is crucial for the bioactivity of the extracts; however, the lack of scientific evidence focusing on the challenges regarding their poor water solubility and the dependence on the temperature and pH environment, as well as poor bioaccessibility, are still limiting factors for the extract implementation in the in vivo studies [47,48]. Therefore, new solutions and technologies have emerged rapidly to promote and improve their bioaccessibility, including extract/component encapsulation [49,50,51,52], the synthesis of biomass-derived nanoparticles [53,54,55,56], nanocarriers, and biofilm preparation [57,58,59]. The identification and structural characterization of each chemical component present in the plant extract is surely beneficial to fully understand the mechanism for the formulation preparation, as well as to predict the underlying potential mechanism of action for in vitro and in vivo systems [60,61].

This review article gathers all the relevant evidence on the beneficial effects and promising health-promoting potential of the pure extracts and isolated phytochemicals derived from citrus by-products and aims to collect and provide the most recent literature on the developments and innovations regarding citrus-based by-products’ incorporation into the pharmaceutical, biomedical, and biological scientific areas. The major focus of this review will be directed toward providing a comprehensive view of the compositions of citrus by-products, such as essential oils, carotenoids, pectins, and phenolic compounds, based on their positions within the citrus fruit tissue, and also highlighting a wide diversity of their possible bioactivities and functionalities. Furthermore, the most recent improvements and developments in citrus by-product utilization, especially regarding the innovations in the pharmacological and biomedical fields, will be also reported, while the recent literature is indicative that nanotechnology could play a crucial role toward the specific drug delivery and bioaccessibility enhancements of versatile plant metabolites and plant-based formulations.

## 2. Structural and Chemical Characteristics of Citrus Fruits By-Products

**Figure 1:**
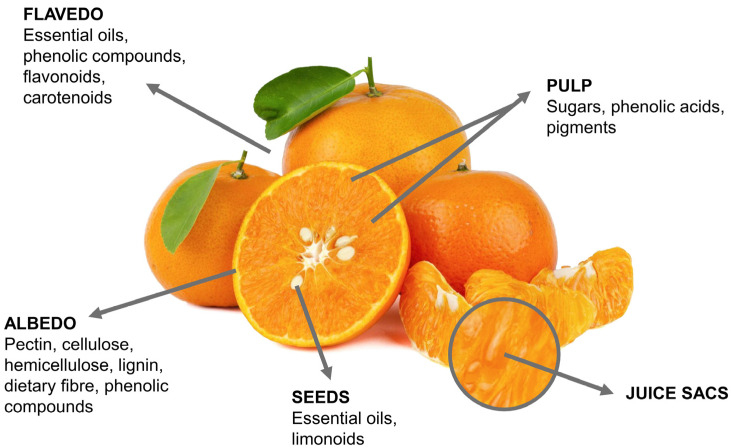
Anatomical and structural characteristics of citrus fruits.

Citrus fruits, like many other agricultural products, are characterized by their agricultural biodiversity [62]. Their physicochemical characteristics, as well as a diversity of chemical compounds, depend on a variety of factors and environmental conditions, such as soil, fertilization, age, position on the tree, maturity, and others [62,63,64,65]. It is interesting that all varieties of citrus fruits, by means of microscopic and macroscopic views, have similar structural and anatomical characteristics. A schematic view of the anatomical characteristics and structural compositions of citrus fruits is presented in Figure 1.

Citrus fruits are widely consumed due to their nutritional qualities and appealing taste and fragrance, and the products of citrus processing mostly include the production of food-grade (jellies, jams, candies, flavoring agents, etc.) and aromatic/cosmetic (essential oils) products [10,66,67]. The generation of significant amounts of waste during citrus fruit processing is a major concerning issue, as the waste represents almost 50% of the fresh fruit mass [66]. The generated waste includes by-products such as peels (the highest percentage of almost 50%), seeds (20–40%), pomace, and wastewater (the residue of spoiled parts of the fruits) [66,67,68].

The outer layer of the citrus fruit consists of the peel, which can be roughly divided into two regions: flavedo (lat. flavus means yellow) and albedo (lat. albus means white) [69,70]. The flavedo region comprises characteristic peel oils and pigments, and the white spongy part of the peel is referred to as the albedo [71]. Although, it is not uncommon that some literature reports refer to flavedo as epicarp and albedo as mesocarp [72]. The flavedo region is covered with a thin layer of cuticle, consisting of natural waxes and continuous polymerized materials [60,69]. The role of the cuticle is mainly protective against microorganism attacks, limit vapors, and water loss, regulating also the exchange of oxygen and carbon dioxide [73,74]. From a chemical point of view, natural waxes are characterized by the presence of long-chain alkanes, fatty acids, aldehydes, and alcohols [68,72], while the polymerized material originates from hydroxylated fatty acids [75]. Below the cuticle and within the flavedo region, pigments and essential oils are present. The citrus pigments are located within the chloroplasts (if green) and in chromoplasts (if yellow, orange, or red color) [62]. The composition and differences in the carotenoid content determine the color of citrus fruits [76]. The green or yellowish-green color of immature citrus fruits originates from the accumulation of lutein and a certain content of chloroplastic carotenoids, such as β- and α-carotene, neoxanthin, and zeaxanthin [76]. However, the orange color formed during the natural ripening of citrus fruits is caused by the increase in the content of colored carotenoids (β,β-xanthophylls) and by a decrease in the lutein concentration [77,78]. The essential oils are found in the oil glands located in the citrus flavedo layers and are defined as fragrant compounds present in the peel. The citrus essential oils consist of volatile compounds in the majority (85–99%) and in lower fractions as non-volatile compounds (1–15%) [79]. Interestingly, it has been reported that essential oils, although referred to as oils, are not typically oils in the chemical sense due to the absence of triglycerides, making this group of components the mixture of terpenes and terpenoids, which give rise to their hydrophobic character [62]. The volatile composition of citrus essential oils includes the highest content of monoterpenes (70–95%); sesquiterpenes; and their oxygenated derivatives (aldehydes, alcohols, esters of carboxylic acids, and ketones) [62,80], with *d*-limonene monoterpene as the major constituent of citrus essential oils [79,80,81]. The non-volatile fraction consists of carotenoids; fatty acids (oleic, linoleic, linolenic, stearic, palmitic, etc.); waxes; flavonoids; and sterols [82].

The albedo layer is considered to be a white and relatively porous layer of the citrus peel, consisting of pectic substances, cellulose, starch, and phenolic compounds [62,83]. The pectins are a complex group of compounds defined as non-starch polysaccharides, mostly consisting of conjugates of D-galacturonic acid; acid groups (methoxy esters); and some neutral sugars (rhamnose, glucose, xylose, arabinose, etc.) formed through α-(1-4)-glycosidic bonds [84,85]. Based on the degree of methylation and acetylation—more precisely, by the number of methoxyl and acetyl groups substituted by the carboxylic acid on the D-galacturonic acid chain—high-methoxyl or low-methoxyl pectins can be formed [84]. Although it is well known that low-methoxyl pectins are used in the food industry due to their gelation characteristics [86], pectins are also studied for their utilization in pharmacy and medicine (cholesterol reduction, drug delivery, immune modulation, etc.) [85]. Organic acids are classified as low molecular weight compounds that play a crucial role in plant metabolism; however, organic acids also exhibit protective and health-promoting activities [65,87]. The most commonly detected organic acids in citrus by-products are citric and malic, while benzoic, oxalic, tartaric, and succinic acids are also present in citrus by-products [88]. The most abundant vitamin in citrus fruits is ascorbic acid, most commonly known as vitamin C, while citrus fruits can also be a good source of folate (vitamin B9) [11,64,89]. Those vitamins have an important role in regulating immune functions with immune-enhancing potential [89]. Both organic acids and vitamins are predominantly found in the juice vesicles, located in the endocarp of citrus fruits [65].

**Figure 2:**
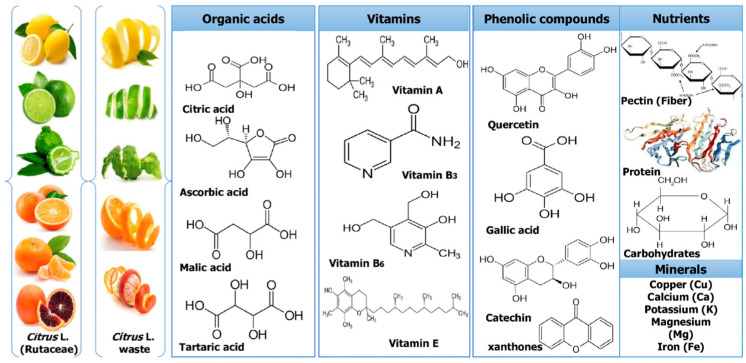
The abundant phytochemicals in citrus fruits and their by-products. Reprinted from ref. [11].

Furthermore, phenolic compounds and flavonoids provide several benefits associated with health-promoting effects [65,90]. The major flavonoids present in citrus peels are hesperidin, narirutin, naringin, and rutin, while the content of each flavonoid depends on the physicochemical characteristics of the cultivated fruit and analyzed citrus variety [91]. The abundant chemical components present in *Citrus* fruits are shown in Figure 2. The citrus endocarp consists of juice vesicles and seeds, and interestingly, few scientific studies have reported the superior activity of citrus seed extracts compared to peel extracts [92,93]. The juice mostly consists of water (85–90%), and the soluble solids include carbohydrates; pigments; vitamins (vitamin C and vitamin B complex); minerals (calcium, potassium, magnesium, copper, and iron); and organic acids, while the pulp consists of the particles insoluble in the suspension of the juice [11,62,88].

The full potential of citrus seed utilization is still an area of interest for the scientific community, as there have been fewer studies dealing with citrus seed valorization and utilization [94]. One study reported the utilization of citrus seeds for fixed oil production, which were enriched with tocopherols, phytosterols, sugars, carotenoids, and minerals [95]. These oils were found to be adequate for soap making.

## 3. Converting Waste into Treasure—Utilization of Citrus By-Products

Recently, waste management has become one of the great concerns globally, and its valorization has created more sustainable and smart waste management solutions. Primarily, waste valorization includes employing different technologies toward obtaining value-added products with a wide spectrum of potential applications. Citrus by-products have been extensively studied due to their rich-bioactive properties, and their valorization enables beneficial gains from both economical and environmental points of view [96,97]. The scientific focus has been placed on innovative extraction methods for obtaining high-quality citrus essential oils [98,99,100,101] and enriched extracts in general [11,94,102,103]. Interestingly, the authors Tunç and Odabaş [99] reported a single-step ohmic heating-assisted extraction/hydrodistillation (OHAE/H) procedure to obtain the simultaneous recovery of essential oils and pectins from lemon waste. The process was optimized to obtain the maximum recovery of both components by response surface methodology. The liquid-to-solid ratio (*w:v*) and extraction/hydrodistillation time (min and voltage gradient (V/cm) were determined as independent variables, while the maximum pectin and essential oil yields were maintained as the dependent variables. The results obtained by the OHAE/H method were compared to the conventional extraction methods, and it was concluded that OHAE/H showed superior performance compared to the conventional methods. Moreover, in the study by Hwang et al. [104], the efficiency of hesperidin and narirutin extraction was investigated by combining pulsed electric field and subcritical water extraction techniques. Firstly, the samples were subjected to pulsed electric field treatments at the strength of 3 kV/cm for the times of 60 and 120 s. Subsequently, the subcritical water extraction was applied under the conditions of temperatures of 110–190 °C for 3–15 min. It was shown that the pulsed electric fields method enhanced the extraction process for obtaining both narirutin and hesperidin, increasing the yields by 22.1% and 33.6%.

Solid citrus waste can be also utilized for the production of animal food. Due to its good nutritional composition containing dietary fibers, lipids, flavonoids, enzymes, vitamins, and carotenoids, citrus waste represents a promising by-product for the production of livestock feeds [105]. The literature reports that citrus pulp (the main residue after juice extraction), citrus molasses (produced by concentrating on the press liquor of citrus peel residue with a high content of sugars), citrus peel liquor (similar to molasses but not as concentrated), and citrus-activated sludge (produced from liquid waste) could be considered as by-product feedstuffs [106]. The nutrient content of citrus by-products mainly depends on the source and variety of citrus fruits, as well as on the type of processing [88]. The main issues in the utilization of citrus by-products for the production of feedstuffs are the low nitrogen content and poor storage, which can lead to the development of mycotoxins [88]. Another valuable utilization of citrus waste is the production of packaging films that meets all the standards of sustainable and biodegradable forms of biopolymers [10,107]. Conventional packaging films are considered an environmental concern due to their poor biodegradable properties, and therefore, new innovative and sustainable solutions are welcomed [107]. An important advantage of using biopolymers derived from plant materials is that those raw materials naturally contain significant amounts of bioactive components that exhibit antioxidant and antimicrobial properties. The matrix of the citrus-based package is pectin, which enables solid support for the production of active packaging films [10]. In the study by Meydanju et al. [108], biodegradable film was prepared from lemon peel waste. Firstly, a composite of lemon waste powder, xanthan gum, and TiO_2_-Ag nanoparticles was produced. The additives enhanced the physicochemical properties of the prepared packaging; more precisely, the xanthan gum addition increased the thickness of the film, as well as the moisture content, due to the presence of -OH functional groups and the possibility of the hydrogen bonds forming. Also, both additives improved the thermal stability of the packaging films, and the antioxidant and antimicrobial properties were enhanced as well.

The application of plant-based and phenolic extracts in the food industry is highly limited due to their poor bioaccessibility, low water, and liquid solubility, while it is well known that bioactive phenolic compounds are extremely sensitive to light, oxidants, and changes in pH conditions and temperatures [47,97,109]. The main challenge presents as overcoming the limiting incorporation of low water-soluble compounds into aqueous-based foods, which directly limits the proper gastrointestinal bioaccessibility. In order to overcome these issues, encapsulation has been employed more frequently to protect bioactive compounds [47,110,111]. The spray-drying and freeze-drying techniques are commonly used methods for obtaining stable encapsulated functional substances, while extrusion methods, coacervation, and emulsification methods have been also applied [110]. In the study by Papoutsis et al. [112], different formulations of lemon by-product extracts combined with maltodextrin-coating agents (maltodextrin, maltodextrin and soybean proteins, maltodextrin, and ι-carrageenan) were prepared by both the spray- and freeze-drying methods. As expected, the formulations exhibited different morphological characteristics and showed an amorphous nature. The highest antioxidant activity was demonstrated with the sample containing lemon waste extract, maltodextrin, and soybean protein prepared by the freeze-drying process. In this case, the problem presented was polyphenol degradation due to freezing and dehydration and grinding the sample after the lyophilization, which could cause polyphenol oxidation. Moreover, polyphenol degradation occurred in the samples encapsulated by spray-drying, and exposing them to high inlet temperatures led to a significant decrease in the contents of the phenolic compounds.

Another interesting case of citrus by-product utilization is bioconversion into biofuels. It is known that the citrus residue contains significant amounts of carbohydrates and fermentable sugars; however, also, high contents of bioactive compounds inhibit the possible fermentation processes [113]. This issue could be overcome by extracting those bioactive molecules to be further used for biosorbents, biogas, and ethanol production by biotransformation. For example, the authors Oberoi et al. [114] used Kinnow mandarin waste to produce and optimize the process of bioethanol production. It has been reported that mandarin peel contains a high content of sugar, cellulose, and pectins and low content of lignin, making their citrus waste a promising substrate for bioethanol production. The process includes enzymatic hydrolysis, where the biomass is digested by enzymes into pentose and hexose sugars, which are used by microbes in the fermentation process.

## 4. Bioactivities of the Individual Groups of Compounds Present in Citrus By-Products

There are numerous published overviews dealing with the valorization of citrus by-products and their potential utilization in the food and cosmetic industries, emphasizing also their health-promoting properties due to the presence of bioactive compounds [1,2,3,4,11,13,97,105]. The present study uses the recent available scientific literature linking citrus by-products to their potential biological, pharmacological, and biomedical applications and includes both the utilization of plant extracts and pure compounds that can be separated from the citrus by-products. Furthermore, a brief overview of the application of nanotechnology in waste management and food science will also be provided.

### 4.1. Waxes and Carotenoids

**Figure 3:**
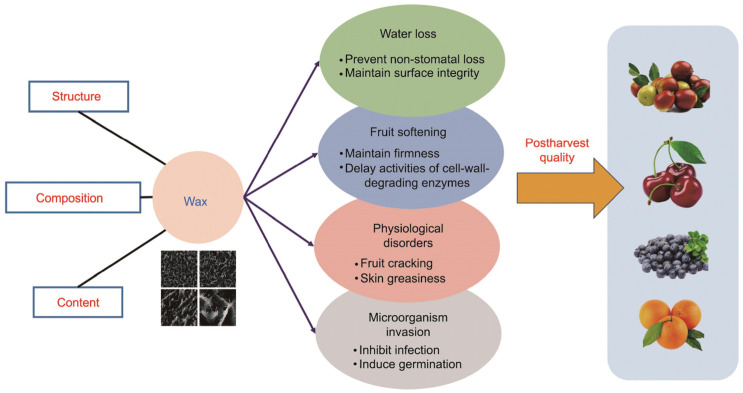
Schematic presentation of the role of cuticular wax in fruits. Reprinted from ref. [115].

Cuticular wax plays an important role in fruit preservation and proper storage, and it is well known that it acts as a natural barrier that protects plants from biological and non-biological stress [73,74]. Also, the structural characteristics, content, and composition of cuticular wax have been found to affect the postharvest storage quality against fruit water loss and softening and could be responsible for the resistance to fruit diseases, as summarized in Figure 3. Waxes are comprised of long-chain fatty acids and their derivates, esters, aldehydes, ketones, primary and secondary alcohols, and triterpenoids [115]. Most of the studies related to the topic of citrus cuticular waxes focused on the synthesis and transcriptional regulation of cuticular wax in citrus fruits. However, the authors Zhu et al. [116] carried out an investigation of the influence of *C. reticulata* cuticular wax on the colony expansion of the fungal pathogen *Penicillium (P.) digitatum* (green mold). The investigation included in vivo and Formvar^®^-based in vitro systems. Finally, it was concluded that the cuticular wax of mandarin fruit impairs *P. digitatum* colony expansion, acting as a physical barrier exhibiting antifungal activity. Furthermore, the content of carotenoids and the phytochemical profile of citrus fruits in general depend on the citrus variety, ripening stage, and the tissue [117]. It has also been reported that citrus fruits contain approximately 120 different carotenoids, classified as xanthophylls and carotenes [117].

The investigation of the carotenoid content separated from *C. reticulata* by-products and its influence on the immuneoxidative status of broiler chickens was carried out by Mavrommatis et al. [118]. The carotenoid-rich extract was prepared, and the chickens were fed a supplemented diet consisting of a freeze-dried formulation containing carotenoid extract and soluble starch. It was demonstrated that carotenoid-supplemented feed exerted inhibitory activity against Gram-positive (*Staphylococcus (S.) aureus*), as well as Gram-negative (*Klebsiella (K.) oxytoca*, *Escherichia (E.) coli*, and *Salmonella (S.) typhimurium*), bacteria. The implementation of the carotenoid content in the supplementation led to alanine aminotransferase and breast muscle malondialdehyde, and the activity of superoxide dismutase increased. Also, several parameters were downregulated, such as catalase, NADPH oxidase 2, interleukin 1β, and tumor necrosis factor. In the study by Barman et al. [119], β-carotene-loaded nanoemulsion was prepared from *C. reticulata* peels with the primary aim of carotenoid bioaccessibility improvement in fruit juice. Firstly, β-carotene was extracted using a mixture of hexane/acetone/ethanol solvents, and the sample was centrifuged, dried, and filtrated for nanoemulsion preparation. The nanoemulsions were prepared by suspending *C. reticulata* extract in hexane, and afterward, surfactants were added (caprylocaproyl polyoxyl-8-glycerides, polyoxyethylene, sorbitan monolaurate, and polyoxyethylene). The hexane was removed by rotary vacuum evaporation. The nanoemulsions were characterized by means of a physicochemical analysis, while the in vitro studies included gastrointestinal and gastric digestion. The results demonstrated that prepared formulations significantly increased the bioaccessibility of β-carotene and retinol activity equivalent in fruit juice. This study offered an alternative to synthetic color as a natural food colorant and, at the same time, provided health-promoting benefits to customers.

### 4.2. Aromatic Compounds—Essential Oils

Citrus essential oils are known as a fragrant mixture of chemical compounds exhibiting versatile activities used in the food, cosmetical, and pharmaceutical industries, as well as in aromatherapy [79]. The involvement of nanotechnology has provided new solutions for developing essential oil-based nanosystems with the aim of bioaccessibility enhancement. Interestingly, the formulation of *C. lemon* essential oil in nanohexosomes was prepared by the group of authors Sedeek et al. [120] for the purpose of antifungal activity investigation. Firstly, *C. lemon*, *C. aurantifolia*, *C. maxima*, and *C. sinensis* essential oils were extracted using hydrodistillation in a Clevenger’s apparatus from powdered peels. The hexosomal dispersions loaded with oils were prepared by the hot emulsification method reported by Abdel-Bar et al. [121]. In addition, the different obtained essential oils were assessed against phytopathogenic fungi (*Rhizoctonia (R.) solani*, *Sclerotium (S.) rolfsii*, *Fusarium (F.) solani*, *Fusarium (F.) oxysporum*, *Fusarium (F.) semtectium*, *Botrytis (B.) cinerea*, and *Alternaria (A.) alternata*), and it was concluded that all the tested essential oils exhibited strong antifungal activity, showing dose-dependent behavior. The *C. lemon* and *C. aurantifolia* essential oils exerted superior antifungal activity compared to the other essential oils, demonstrating the complete inhibition of *F. solani*, *S. rolfsii*, and *F. oxysporum* growth, while the *C. lemon* essential oil showed exclusive antifungal activity against *A. alternata* mycelial growth. Furthermore, the nanohexosomal formulation was prepared by using the best-performing sample of *C. lemon* essential oil, and it was shown that the nanohexosomal formulation completely inhibited mycelial growth of *F. solani* fungi at the applied concentration of 600 µL/mL, while the complete inhibition of *S. rolfsii*, *A. alternata*, and *F. oxysporum* was achieved at the concentration of 800 µL/mL. A moderate inhibitory effect was observed against *R. solani*, *B. cinerea*, and *F. semtectium*, with determined IC_50_ values of 416, 549.4, and 534 µL/mL, respectively. The authors Feng et al. [122] reported the potential hypercholesterolemia and hepatic steatosis preventive effects in male Sprague–Dawley rats on a high-fat diet. The essential oil of *C. reticulata* peel was obtained by the subcritical fluid extraction method, and limonene was determined to be the dominant component present in the essential oil formulation, followed by γ-terpinene and β-myrcene. The study combined biochemical analysis, lipidomics, and genes to investigate the hepatic steatosis and cholesterol improvements in high-fat diet rats. The high-fat diet in rats induced an increase in fat mass, liver weight, and hepatic lipid deposition with high serum and hepatic triacylglycerol levels. By introducing citrus essential oil as a food supplement, the total levels of the fatty acids, triacylglycerol, and cholesteryl ester classes in liver tissue significantly decreased, while the downregulation of lipogenesis-related genes and upregulation of bile acid-related genes was observed. The potential physiological stress amelioration and anti-inflammatory effects of *C. depressa* Hayata essential oil were reported by Asikin et al. [123]. The essential oil of *C. depressa* Hayata was extracted from the citrus pulp by applying hydrodistillation with a Clevenger-type apparatus. The GC–MS phytochemical profile analysis confirmed the presence of two dominant aromatic compounds, limonene and γ-terpinene. The influences of citrus essential oil on the neurological stress levels in nine healthy female panelists were monitored through electrocardiography (ECG) and electroencephalography (EEG) measurements, while the anti-inflammatory activity was assessed by nitric oxide (NO) and interleukin-1β inhibitory assays. By suppressing proinflammatory markers, the citrus essential oils showed promising anti-inflammatory potential, while the results of the EEG and ECG showed improvements in mental focus and stress reduction activity upon citrus essential oil inhalation.

A summary of the literature reporting the bioactivities of citrus-based essential oils is shown in Table 1.

**Figure 4:**
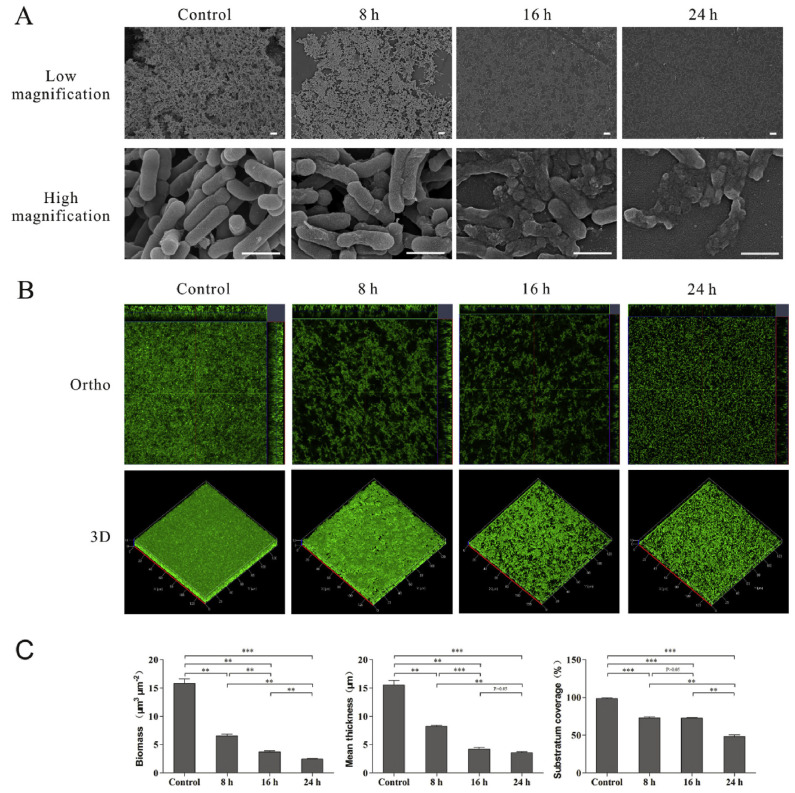
*L. monocytogenes* biofilm investigation by using SEM and CLSM imaging after 8, 16, and 24 h of Huyou essential oil treatment; the control sample represents untreated biofilm cells: (**A**) SEM imaging, (**B**) CLSM imaging, and (**C**) COMSTAT analysis; bars represent the mean values (mean ± SD; n = 3), “∗” pointed to significantly enrichment. *p* < 0.001 were labeled as “∗∗∗”, *p* < 0.01 were labeled as “∗∗”, and *p* < 0.05 were labeled as “∗”. Reprinted with permission from ref. [126]. Copyright 2019 Elsevier.

Furthermore, the antimicrobial efficacy of citrus-based essential oils against foodborne pathogen *Listeria monocytogenes* (*L. monocytogenes*) was demonstrated in the study by Guo et al. [126]. Gram-positive bacteria *L. monocytogenes* is a highly adaptable pathogen that causes listeriosis, a life-threatening infection, and it is especially dangerous if the central nervous system is affected [141]. The essential oil from the *C. Changshan-huyou* Y.B. Chang (Huyou) species was extracted from peels by the steam distillation procedure using water as the solvent. The antimicrobial and antibiofilm ability of citrus essential oil was investigated against the *L. monocytogenes* pathogen, while the antilisterial mechanism was studied at the microscopic (SEM and TEM) (Figure 4) and molecular levels (RNA-seq analysis).

The results of the antimicrobial activity of Huyou essential oil against *L. monocytogenes* showed dose-dependant antimicrobial activity when comparing treatments of pathogens with the 1xMIC (minimum inhibitory concentration), 0.25xMIC, and 0.125 MIC. The study also discussed the changes in the physical morphology of *L. monocytogenes* biofilms when treated with 1xMIC for 8, 16, and 24 h by using scanning electron microscopy (SEM) and confocal laser scanning microscopy (CLSM) analyses. As shown in Figure 4, the control sample demonstrated intact and complex structures, and upon the addition of Huyou essential oil, the destruction of the biofilms was observed. The most significant differences were observed after 16 and 24 h of treatment, where lysis and the death of the cells in the biofilms of *L. monocytogenes* were observed. When comparing the results of three different methods, such as SEM, CLSM, and COMSTAT, the authors concluded that, in the early stage of treatment (8 h), the predominant effect of rapid detachment of the biofilm was more likely to occur, while, in late stages (16 and 24 h), cell death might be the major effect to eradicate the rest of the biofilm. This study showed the great potential of citrus-based essential oils to be used as a natural food preservative for shelf life extension.

### 4.3. Pectins

**Figure 5:**
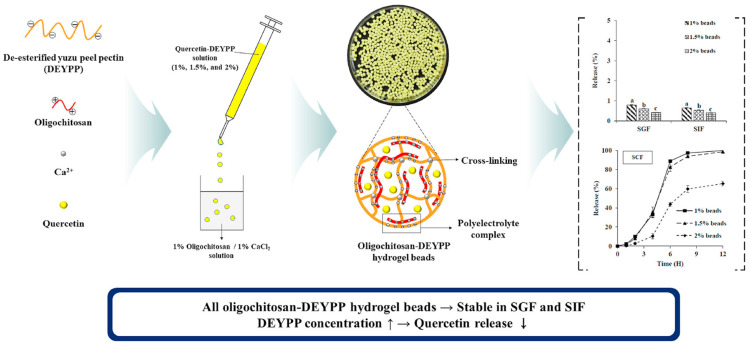
Schematic illustration of the hydrogel beads used as a quercetin delivery system for the colon target. Reprinted with permission from ref. [146]. Copyright 2020 Elsevier.

The importance of pectins in the food industry is widely known; however, this group of polysaccharides has found their place in a variety of human applications, such as in the pharmaceuticals, cosmetics, drug delivery, and biomedical fields [86,141,142]. The versatile application of pectic biopolymers is enabled due to their structural diversity and chemical complexities, as well as the possibility of structural modifications [143]. The recent literature reports dealing with the bioactivities of citrus-based pectins are listed in Table 2. Recently, there has been an increase in interest in pectic biopolymers, mainly for their wide spectrum of bioactivities, and recently, pectic oligosaccharides have been evaluated for their promising prebiotic activity [144]. In addition, Zhang et al. [145] obtained pectin oligosaccharide fractions by the controlled degradation of citrus peel pectin. Three different oligosaccharides were prepared by adjusting the concentration of trifluoroacetic acid or H_2_O_2_ at the appropriate pH value, producing pectin oligosaccharides of variable molecular weight ranging from <2000 Da, 2000 to 3000 Da, and 3000 to 4000 Da. The results demonstrated a high prebiotic activity (pectic oligosaccharides obtained by H_2_O_2_ oxidation; 3543 Da) for *Bifidobacterium (B.) bifidum* and moderate activity against the *Lactobacillus (L.) paracasei* bacterial species. This study showed the enormous prebiotic potential of citrus-based pectic oligosaccharides; however, the greatest challenge remains to be overcome, as the human gastrointestinal tract includes complex pH-dependent processes and the presence of different enzymes that could affect in vivo digestion and bioaccessibility. Another interesting application of pectic biopolymers is their utilization as a carrier for drug delivery systems. The authors Lee and Chang [146] prepared quercetin-loaded hydrogel beads for the colon target, produced by deesterified pectin from yuzu (*C. junos*) peel and oligochitosan. A schematic illustration presenting the quercetin-loaded hydrogel beads preparation procedure and potential application in targeted therapy for colon cancer is shown in Figure 5.

For the purpose of the study, low-methoxyl pectin (DEYPP) was produced by deesterification with pectin methylesterase treatment, which is used for quercetin-DEYPP preparation. The hydrogel beads were prepared by dropping quercetin-DEYPP solution into a calcium chloride solution (1% *w*/*w*; pH = 6) containing oligosaccharide (1% *w*/*w*). Previously, cumulative quercetin release exposed to the simulated gastric fluid and intestinal fluid was very low (below 1%), and quercetin-loaded hydrogel beads significantly improved the bioaccessibility of quercetin in simulated colonic fluid (65.37–99.54%), which demonstrated the great efficiency of the developed quercetin drug delivery system for colon targeting. Furthermore, an example of citrus pectin-based drug delivery was reported by Jacob et al. [147], introducing pectin nanoparticles fabricated by ionotropic gelation using Mg^2+^ as a divalent cross-linker with the affinity of linking to the reactive carboxyl groups. Three different samples of nanoparticles were prepared as follows: high-methoxyl, low-methoxyl, and amidated low-methoxyl pectins. The cell viability on THP-1 (human leukemia monocytic cell lines) confirmed their excellent biocompatibility and potential application as a nanocarrier for oral drug delivery.

**Table 2:** Summary of the literature reporting bioactivities of pectins extracted from citrus by-products of the past six years (2018–2023).

Source	Formulation/Chemical Analyte	Bioactivity	Testing Subjects	References
*C. unshiu* peel	Extracted pectin (pH = 3; precipitation using 95% ethanol)	Antioxidant activity	Total phenolic content (TPC), DPPH^•^, ABTS^•+^, FRAP assay, ferrous ion chelating activity	[148]
Citrus peel ^1^	Commercially purchased pectins; pectin-capped copper sulfide nanoparticles (pCuS NPs)	Antifungal activity	In vitro on *Candida albicans*	[149]
Citrus peel ^1^	Pectin oligosaccharide fraction obtained by controlled chemical degradation of citrus peel pectin (commercial)	Prebiotic activity	In vitro on probiotic strains *Bifidobacterium* spp. and *Lactobacillus* spp./	[145]
*C. unshiu* Marc. waste (remains from the canning processes)	Depolymerized RG-I-enriched pectin	Prebiotic activity	In vivo on male mice; Total serum cholesterol and triacylglycerol concentrations; *Bacteroide thetaiotaomicron*, *Bifidobacterium Longum*	[150]
Citrus (lime/lemon) peel ^1^	High methoxylated citrus pectin nanoparticles (HMP-NPs), low methoxylated citrus pectin nanoparticles (LMP-NPs), and low methoxyamidated citrus pectin nanoparticles (AMP-NPs)	Oral drug delivery	In vitro cell viability tests on THP-1 (human leukemia monocytic cell line) cell line	[147]
Yuzu (*C. junos*) peel	Extracted pectin (pH = 3.5; precipitation using 95% ethanol)/de-esterification of pectin/oligochitosan/quercetin hydrogel beads preparation	Drug delivery/quercetin delivery system for the colon target	In vitro release study using simulated gastric, intestinal, and colonic fluids	[146]
*C. reticulata* peels	Extracted pectin (UAE ^2^; ammonium oxalate-oxalic acid—pH = 3.4; precipitation using 96% ethanol)	Potential antitumor activity	In vitro on the normal human embryonic kidney (HEK293) cells and colon cancer (HT29) cells	[151]
Lemon and lime peel ^1^	Commercially purchased pectins	Anti-colitis activity/anti-inflammatory effect	In vivo on male C57BL/6 mice	[152]
*C. sinensis* peel (IntegroPectin)	Commercially purchased pectins/hesperidin-rich citrus pectin	Prevention and therapy of COVID-19	Computational studies: molecular model of the 3-chymotrypsin-like protease (3CLpro) structure of the SARSCoV-2	[153]
Citrus peel ^1^	Citrus pectin oligosaccharides obtained by H_2_O_2_ degradation	Hypocholesterolemic effects	In vivo on male C57BL/6 mice	[154]
Grapefruit peel (IntegroPectin) ^1^	IntegroPectin isolated by freeze-drying of water-based extract	Cardioprotective effects	In vivo on male Wistar rats	[155]

^1^ Species not specified; ^2^ UAE—ultrasound-assisted extraction.

### 4.4. Phenolic Compounds

Natural phenolic compounds have been studied extensively for their essential role in plant protection, as well as for their beneficial effects on human health. It is well known that citrus by-products contain substantial contents of different phenolic compounds in the forms of acids and flavonoids, which have recently become the great subject of studies as natural antioxidants [156,157]. The representative bioactive compounds for the citrus family are flavanone aglycones (hesperetin, naringenin, and eriodictyol); flavone and flavonol aglycones (kaempferol, quercetin, apigenin, and diosmetin); flavanone-7-*O*-glycosides (eriocitrin, hesperidin, naringin, narirutin, poncirin, and didymin); and polymethoxyflavones (PMFs; nobiletin, tangeretin, and sinensetin) [62,91,158]. A summary of their bioactivities is listed in Table 3.

**Figure 6:**
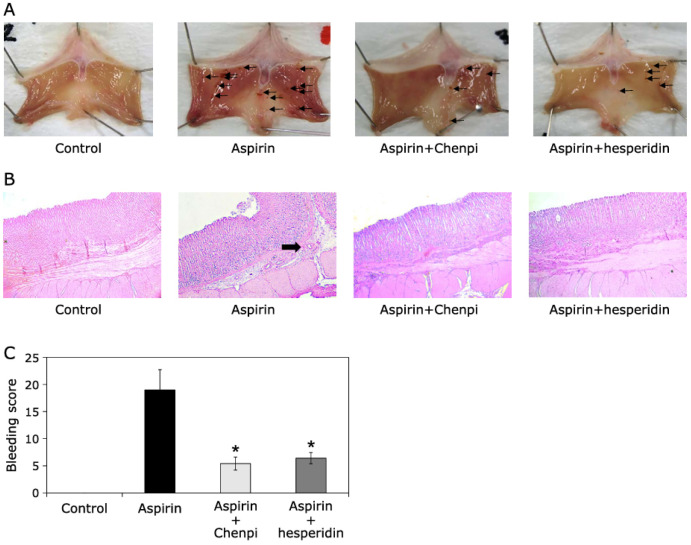
(**A**) Effects of citrus extract and hesperidin on the gastric mucosa in aspirin-induced lesions in rats. (**B**) Photomicrographs showing the macroscopic appearance of the stomach. (**C**) Gastric bleeding score in rats by applying different formulations. The data represent the mean ± SD (*n* = 5, per group). Statistical analysis was carried out with the Steel–Dwass test; * *p* < 0.05. Reprinted from ref. [159].

The great potential of citrus-based extracts lies in their health-promoting ability, exhibiting a wide spectrum of bioactivities, such as antioxidant, anti-inflammatory, and antiproliferative activity, against cancer. Interestingly, Shimamura et al. [159] studied the protective effects of hesperidin-rich extract obtained from *C. unshiu* (Chenpi) peel and commercially supplied hesperidin on aspirin-induced oxidative damage in rats. One of the major possible side effects of aspirin prescription and consumption is the possibility of peptic ulcer formation, which represents a serious gastrointestinal disease [160]. The citrus extract was obtained by reflux extraction, and a HPLC analysis confirmed the abundant presence of hesperidin in the extract sample. In order to evaluate the inhibitory effects of citrus extract and hesperidin on DNA oxidative damage in the stomach, kidney, and liver, the formamidopyrimidine DNA glycosylase (Fpg)-modified comet assay was applied. Also, the in vivo studies included five-week-old male ddY mice for evaluating analgesic activity and nine-week-old male Wistar rats for assessing oxidative damage. As demonstrated in Figure 6, the inhibitory effects of citrus extract and hesperidin were obvious by studying the aspirin-induced oxidative gastric mucosal injuries and by the reduction of the 8-oxoG content (content increases by oxidative stress) when the combined drug was administered.

Finally, the study indicated the protective effects of citrus extract and hesperidin in aspirin-induced damage, while the pharmacological action of aspirin did not change significantly. The important role of hesperidin in gastrointestinal health was also reported by Sharaf et al. [161]. Hesperidin was extracted from *C. uranium* peel by Soxhlet extraction with petroleum ether and methanol, while the crystallization of pure hesperidin was done with 6% glacial acetic acid (pH = 3–4). The isolated hesperidin was investigated for anti-*Helicobacter (H.) pylori* activity, which is also the main contributor to the occurrence of chronic gastritis and peptic ulcers, also increasing the risk of gastric adenocarcinoma [162]. The progressive reduction in urease activity by hesperidin and urease inhibition kinetic analyses indicated the anti-*Helicobacter pylori* activity of hesperidin by competitive mode in a time-dependent manner. Also, the in situ visualization of antimicrobial activity by laser scanning confocal microscopy (LSCM) demonstrated that hesperidin administration led to amino acid leakage from bacterial cells, while scanning electron microscopy (SEM) demonstrated the interaction of hesperidin and bacterial cells causing cell disruption and leakage of the cytoplasmic content. Furthermore, the molecular docking and simulation of the inhibitory effect of hesperidin (urease–ligand) on *HpUre* enzyme through slow-binding inhibition indicated the possible formation of hydrogen bonding, van der Waals, and alkyl interactions with important residues on enzyme *HpUre*-active sites. The bioavailability assays indicated the high potential of hesperidin for oral usage. The beneficial effects on the gastrointestinal system were also reported by applying the Ougan peel extract enriched with nobiletin, tangeretin, and 5-demethylnobiletin compounds exhibiting antitumor activity against gastric cancer cell lines [163], while protective and enhanced anticancer effects of orange peel extract and naringin in the doxorubicin treatment of esophageal cancer cells in a mice model were reported by Tajaldini et al. [164]. The anti-inflammatory effects of citrus-based phenolic compounds are also reported in Table 3. The combination of naringin obtained from *C. maxima* (Burm.) extract and sericin from *Bombyx mori* was investigated for the potential treatment of psoriasis by Deenonpoe et al. [165]. With the assumption that skin inflammation via proinflammatory cytokines is associated with the pathogenesis and clinical manifestation of psoriasis, the inhibitory effect of naringin/sericin drugs on the production of proinflammatory cytokines (TNF-α, IL-6, IL-23, and IL-12p40) and the expression of mRNA of the human peripheral blood mononuclear cells from psoriasis patients were investigated. The study demonstrated the successful dose-dependent formulation of naringin/sericin for downregulating the proinflammatory cytokines related to the inflammation mechanism in psoriasis pathogenesis.

**Table 3:** Summary of the literature reporting bioactivities of different (poly)phenolic compounds extractable from citrus by-products (2018–2023).

Source	Chemical Analytes	Bioactivity	Testing Subjects	References
**Citrus by-products**				
Finger lime peels ^1^	Dominant phenolic acids: malic, citric, and quinic acid/phenolic compounds: neohesperidin, α-glucosyl hesperidin, (7S,8S)-4,7,9,9′-tetrahydroxy-3,3′-dimethoxy- 8-4′-oxyneolignan-9′-*O*-D-glucopyranoside, lyoniresinol 9′-*O*-glucoside and poncirin	Antioxidant, anti-inflammatory effect, neuronal cell protection	Antioxidant: DPPH^•^, ABTS^•+^, FRAP, ORAC/anti-inflammatory: in vitro on BV-2 (mouse microglial) cells and NO release analysis	[166]
Citrus (*C. lumia* Risso) albedo extract (peel and pulp)	Dominant phenolic acids: chlorogenic and ferulic acids/flavonoids: hesperidin and eriocitrin	Antioxidant and cytoprotective activity	Antioxidant: FRAP, TEAC, DPPH^•^, ORAC, β-Carotene bleaching/cell viability on lymphocytes (lactate dehydrogenase (LDH) activity)	[167]
*C. unshiu* (Chenpi) peel	Dominant flavonoid: hesperidin/Hesperidin (commercial product)	Analgesic activity and gastroprotective effect	In vitro on gastric tissue/in vivo on male ddY mice	[159]
*C. amblycarpa* peels and leaves	Phenolics: quercetin, rutin, and ɣ-aminobutyric acid (GABA)	Antihypertensive effects	ACE Inhibitory Activity Assay	[168]
Ougan peel extracts ^1^	Flavonoids: nobiletin, tangeretin, and 5-demethylnobiletin	Antitumor activity	In vitro on gastric cancer cell line AGS, BGC-823 and SGC-7901/in vivo BALB/c nude mice	[163]
*C. reticulata* Cv. Suavissima peel extract	Flavonoids: nobiletin, tangeretin, and 5-demethylnobiletin	Anti-inflammatory effect	In vitro on BV-2 (mouse microglial) cells and NO release analysis, JAK2 inhibitor Ruxolitinib and the STAT3 inhibitor Stattic	[169]
*C. reticulata* Blanco, *C. grandis*, *C. reticulata* c.v. Kinnow, *C. limetta,* and *C. sinensis* peel extracts	Dominant flavonoids: hesperidin, naringin, quercetin, rutin, apigenin, nobiletin, tangeretin	Antioxidant activity, anti-inflammatory effect, neuroprotective effect	Antioxidant: DPPH^•^ and ABTS^•+^ assay/Anti-inflammatory: protein denaturation assay (bovine serum albumin protein denaturation)/neuroprotective: Acetylcholinesterase inhibition assay	[96]
*C. japonica* var. Margarita peel	Detected phenolic acids: *p*-hydroxybenzoic acid, vanillic acid, protocatechuic acid, chlorogenic acid, sinapic acid, gallic acid, ferulic acid, caffeic acid	Antioxidant and antimicrobial activity	Antioxidant: DPPH^•^/Antimicrobial: *E. coli*, *Salmonella (S.) typhimurium*, *S. aureus* and *Pseudomonas (P.) aeruginosa*	[170]
*C. sinensis* (navel orange)	Hydroethanolic extract, naringin, naringenin	Hepatopreventive activity	In vivo on male Wistar rats; histopathological investigation and immunohistochemical detection of p53, Bax, Caspase-3, and Bcl-2	[171]
*C. reticulata* peel	Extract (major components): hesperidin, nobiletin, narirutin, tangeretin, and sinensetin	Antiobesity- related effects.	In vitro on 3T3-L1 mouse preadipocytes	[172]
Ten citrus samples	Detected components: nobiletin, quercetin, diosmin, naringenin, hesperidin, hesperetin, rutin	Anti-estrogenic and anti-aromatase activity/antitumor activity	In vivo on immature female Swiss albino mice/in vitro on MCF-7 and T47D (breast cancer lines), as well as the normal human HFB4 cells	[33]
*C. unshiu* peel	Detected components: rutin, naringin, hesperidin, poncirin	Anti-inflammatory and antioxidant activity	In vitro on RAW 264.7 macrophages (originating from Abelson leukemia virus)	[173]
14 Chinese cultivars (mandarins, oranges, pummelos, hybrids, citrons, kumquats)	Detected components: eriocitrin, naringin, hesperidin, didymin, poncirin, naringenin, hesperetin, sinensetin, nobiletin, tangeretin, and 5-*O*-demethylnobiletin	Antioxidant activity, and effects on intestinal microbiota	Antioxidant: DPPH^•^, ABTS^•+^, FRAP, CUPRAC/a-Glucosidase inhibition assay/bile salt binding capacity determination assay/investigation on fecal samples/in vitro on simulated intestinal fermentation	[13]
Sour orange, sweet orange, and lemon peels ^1^	Dominant phenolic acids: *o*-coumaric acid, benzoic acid, ellagic acid, *p*-Hydroxybenzoic acid/flavonoids: myricetin, naringin, quercetin	Probiotic and symbiotic activity (*Acidophilus-bifidus-thermophilus* (ABT)-Type Synbiotic Yoghurt)	Antioxidant: DPPH^•^/antibacterial: *S. aureus*, *Bacillus (B.) subtilis*, and *E. coli*	[174]
*C. limetta* peel	Hesperidin-rich ethanol extract	Management of the rheumatoid arthritis	In vivo on Charles foster rats and Swiss albino mice	[175]
**Individual components**				
*C. sinensis* L. Osbeck peel and pulp	Hesperidin-rich extract	Antioxidant and antidiabetic activity	Antioxidant: DPPH^•^, ABTS^•+^, iron chelating activity/in vitro α-Amylase inhibition assay	[176]
Commercial product	Hesperetin and quercetin	Drug delivery	In vitro on MDCK II (Madin-Darby canine kidney cells) cell viability	[177]
*C. uranium* peel	Hesperidin	Anti-*Helicobacter pylori* activity	In vitro on human *H. pylori strains*/urease inhibition assay/molecular docking	[161]
*C. reticulata* peel	Hesperidin	Antihyperglycemic, antihyperlipidemic, and antioxidant activity	In vivo on male Wistar rats/biochemical assay and histological investigation	[17]
Commercial product	Hesperetin	Treatment and prevention of cardiovascular diseases	Ex vivo on porcine coronary arteries and human coronary artery smooth muscle cells	[178]
Commercial product	Hesperidin	Antitumor activity	In vivo on male-specified pathogen-free C57BL/6N mice/in vitro on Lewis lung carcinoma (LLC) cells	[179]
Commercial products	A mixture of hesperidin and naringenin	Treatment and prevention of cardiovascular diseases	In vivo and ex vivo on male Wistar rats and aortic rings	[180]
Commercial product	Hesperidin	Antitumor activity	In vitro on PC3 and DU145 (human prostate cancer) cell lines	[181]
Commercial product	Hesperetin and naringenin	Antitumor activity	In vitro on MIA PaCa-2, PANC-1, SNU-213 (pancreatic cancer cell lines), Detroit 551 (skin fibroblast), and human umbilical vein endothelial cells (HUVECs)	[182]
*C. sinensis* var. Valencia peel	Naringenin	Hepato- and renoprotective effects	In vivo on male Wistar rats/histological investigation of the liver and kidney tissues	[183]
Commercial product	Naringenin	Anti-proliferative effect., wound healing	In vitro on human A549 lung cancer cells	[184]
Commercial product	Naringenin, nobiletin, and hesperidin	Treatment of optic nerve injury, neuroprotective	In vivo on 6-weeks-old C57BL/6J mice/in vitro on HEK293T (human embryonic kidney cells) cells	[185]
Combination of commercial products (naringin and doxorubicin), orange peel ^1^	Combination of naringin, doxorubicin, and orange peel extract	Antitumor activity	In vivo on mice models/in vitro on YM1 (human esophageal squamous cancer cell line)/	[164]
*C. junos* Tanaka peel	Naringin	Preventive effect on pulmonary damage	In vivo on male 7-week-old BALB/c mice/in vitro on NCI-H460 (the human lung carcinoma cell lines)	[186]
*C. maxima* (Burm.) Merr peel	Naringin crystals + sericin	Treatment of psoriasis	In vitro on isolated human peripheral blood mononuclear cells, investigation on proinflammatory cytokines (TNF-α, IL-6, IL-12p40, and IL-23)	[165]
Commercial product	Narirutin	Antitumor activity	In vitro on PC-3 (prostate carcinoma and HEK-293 (embryonic kidney) cell lines	[187]
Grapefruit ^1^ peel	Narirutin-rich fractions	Neuroprotective effect (cerebral ischemia/reperfusion injury)	In vivo on male Wistar rats	[188]
Commercial product	Poncirin	Antidiabetic activity	PTP1B inhibitory assay, α-Glucosidase inhibitory assay, HRAR inhibition assay/in vitro on C2C12 cell (skeletal muscle cells) line	[189]
Commercial product	Poncirin and isosakuranetin	Beneficial effects on gut microbiota	In vivo on thirty C57Bl/6J male mice/fecal microbiota	[190]
*C. sinensis* peel	Rutin	Antimicrobial activity	In vitro on *Aeromonas (A.) hydrophila* strains	[191]
Citrus peel ^1^	Tangeretin	Antitumor activity	In vitro on MCF-7 and MDA-MB-231 (breast cancer) cell lines	[34]
Commercial product	Diosmetin	Antihypertensive effects	In vivo on adult Sprague–Dawley rats/in vitro: vascular pathway inhibitors	[192]
Commercial product	Diosmetin and diosmin	Anti-inflammatory effect on atopic dermatitis	In vivo on six-week-old female SKH-1 hairless mice/in vitro: RBL-2H3 (basophilic leukemia) cell line	[193]

^1^ Species not specified.

## 5. Citrus By-Products Formulations with Enhanced Bioactivities

**Figure 7:**
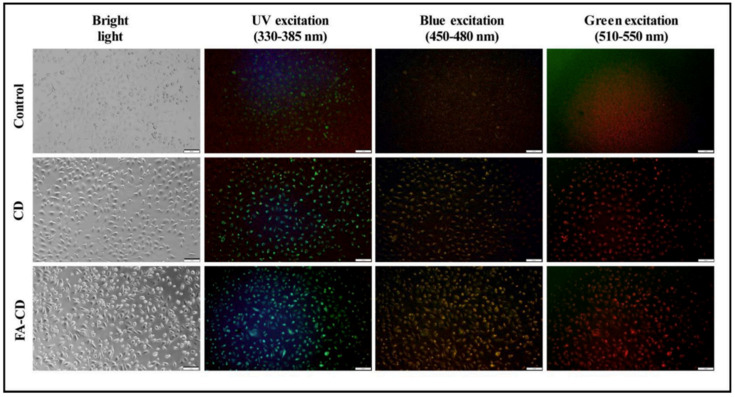
Bioimaging of pure CQDs (designated as CD in the figure) and CQDs conjugated with folic acid (designated as FA-CD) in MCF-7 cell lines. Reprinted with permission from ref. [203]. Copyright 2020 Elsevier.

The biomass-derived compounds are known for their health-promoting properties, and there is a rising trend in waste and by-product valorization to obtain value-added products with a wide spectrum of applications [2,11,194,195]. As it was already demonstrated in Table 1, Table 2 and Table 3, the beneficial effects of different citrus by-products on human health are not disputable; however, poor bioaccessibility is a crucial and limiting factor for successful in vivo applications. Therefore, new and innovative ideas with the implementation of nanotechnology brought about some new solutions in bioaccessibility and bioactivity enhancements (Table 4). The preparation of silver nanoparticles (AgNPs) from citrus (*C. tangerina*, *C. sinensis*, and *C. limon*) peel extract was reported by Niluxsshun et al. [196]. Firstly, citrus peel extracts were prepared by boiling peels in hot water, and afterward, a solution of AgNO_3_ was added to the flask when a golden colloidal suspension was formed. The structural and morphological analyses confirmed the presence of AgNPs in sizes of 10–70 nm, containing different morphological characteristics of nanoparticles. The presence of natural antioxidants, flavonoids, phenolic acids, and other phenolic compounds could act as a reducing agent, leading to the formation of silver nanoparticles. The AgNPs were investigated for antimicrobial activity against the Gram-negative bacteria *E. coli* and the Gram-positive bacteria *S. aureus*, and the results showed the superior antimicrobial activity of orange-based AgNPs on both bacteria strains. Also, it is expected that the bioactivity of nanoparticles is dose- and size-dependent [197], and it is assumed that silver potentially interacts with thiol groups of proteins on cell membranes, causing respiration blocking, which leads to cell death. Another example of AgNP synthesis by using citrus by-products for the purposes of antimicrobial investigation was reported by Alkhulaifi et al. [198]. In this study, *C. limon* peels were used for the synthesis of AgNPs, which were formed by the addition of a AgNO_3_ solution. Again, a possible explanation for the AgNPs formation was the reduction of Ag^+^ ions to silver nanoparticles in the presence of phenolic compounds, and the AgNPs demonstrated spherical- and rod-like-shaped morphologies. The antimicrobial activity investigation was carried out on *Acinetobacter (A.) baumannii*, *S. typhimurium*, *E. coli*, *Pseudomonas (P.) aeruginosa*, *S. aureus*, and *Proteus (P.) vulgaris* human pathogenic bacteria. The results indicated the good performance of AgNPs against the Gram-negative (*E. coli*, *S. typhimurium*, and *P. aeruginosa*) and Gram-positive (*S. aureus*) bacteria. Also, the cell viability on the MCF-7 (human breast cancer) and HCT-116 (human colon carcinoma) cell lines was evaluated, showing dose-dependent behavior. The good antimicrobial performance was potentially explained by four possible mechanisms: (1) the interactions of AgNPs with the cell membrane, altering the membrane permeability and perturbation of respiratory chain enzymes; (2) the gradual diffusion of AgNPs into the cells, leading to the conjugation of nanoparticles to DNA and causing adverse effects on the enzyme activity and the transcription processes; (3) the leakage of subcellular components as the interaction of AgNPs and plasma membrane were formed; and (4) the generation of free radicals [199,200]. Recently, biomass-derived carbon quantum dots (CQDs) are gaining attention due to their biocompatibility and versatile physicochemical and optical properties. By definition, CQDs are fluorescent carbon (zero-dimensional) nanoparticles possessing small size, low toxicity, controllable solubility, and tunable light-emitting properties. Therefore, all of these properties allow CQDs a wide spectrum of applications in bioimaging, biosensing, catalysis, and theranostics [201,202]. The authors Gudimella et al. [203] reported a green synthetic procedure for obtaining CQDs from citrus peel and the conjugation of CQDs with folic acid. A structural analysis confirmed the presence of nanoparticles of sizes 4.6 ± 0.28 nm, while fluorescence spectroscopy indicated that CQDs exhibited multiple colors at different excitation wavelengths. The biocompatibility of the CQDs was assessed on the L929 (mice fibroblasts) cell lines, and the CQDs conjugated with folic acid exhibited low cytotoxicity, showing good biocompatibility. The bioimaging of cancer cell lines was successfully studied on breast cancer (MCF-7) cell lines, as shown in Figure 7. The CQDs were introduced to MCF-7 cell lines and were illuminated by bright light, UV light (330–385 nm), blue (450–480 nm) light, and green (510–550 nm) light. It was demonstrated that MCF-7 cell lines treated with CQDs conjugated with folic acid exhibited brighter fluorescence emission compared to pure CQDs. It was reported that folic acid conjugation could produce a stronger fluorescent signal, which was probably caused by the enhanced cellular uptake of CQDs conjugated with folic acid in the cancer cell lines.

Similar results were obtained in the study by Šafranko et al. [204], where cellular imaging was investigated for the MCF-7 cell lines; however, specific antitumor activity against CFPAC-1 (ductal pancreatic adenocarcinoma) was determined. The CQD nanoparticles were prepared by a hydrothermal procedure from *C. clementina* peels and amino acids (Gly and Arg), and their application in Fe^3+^ ion sensing and bioimaging was determined, also exhibiting specific antitumor and antioxidant activities. A literature overview of innovative synthetic approaches for obtaining citrus by-product-based formulations is listed in Table 4.

## 6. Conclusions and Final Remarks

The enormous amount of waste and by-products generated during citrus processing is of great concern from both economical and environmental points of view. There are many valuable contributions dealing with the valorization of these by-products, converting them into value-added products with potential applications in the food, cosmetic, and pharmaceutical industries. The major focus has been on the promising bioactivities of different citrus by-products and their beneficial effects on human health, and this discovery offers new alternatives for safer, healthier, and sustainable product development. Although citrus by-products can be considered a valuable and natural source of bioactive compounds, the limiting factors for in vivo applicability are the poor bioaccessibility and solubility of different phenolic compounds and antioxidants, as well as sensitivity to light, pH, humidity, and heat. This challenge can be overcome by forming stable nanoemulsions and different formulations on a nanoscale that enhances the bioactivity, as well as the bioaccessibility, of the active substances. Furthermore, one of the major concerns is the lack of evidence for efficient citrus-by-product utilization/extraction at a larger industrial scale, as, currently, the available literature reports the extraction procedures on a laboratory scale and, in limited cases, on a pilot scale. Also, the extraction of bioactive compounds has limitations by means of low extraction yields of individual compounds or groups of bioactive compounds, and it is certainly questionable that these amounts can satisfy the demands of the different industries.

As was discussed in this review article, different citrus-based by-products show enormous potential in pharmaceutical and biomedical fields. The individual citrus-based compounds, mixtures such as essential oils, and extracts exhibit a diversity of bioactivities, including antitumor, antimicrobial, antiviral, antidiabetic, antioxidant, and other beneficial activities with health-promoting abilities. The investigation of the antitumor ability of different citrus-based by-products is an indisputably emerging trend in the pharmacological and biomedical domains; however, the major limiting factor for applying these formulations is often a lack of knowledge regarding the antitumor mechanisms of these by-products. In order for these by-products to be applied as an alternative to synthetic drugs in chemotherapy or in cancer target therapy, future studies should be more focused on the mechanisms of action to fully understand their antitumor activity, as well as on increasing their specific targeting properties for tumor cells. Furthermore, citrus-based by-products have enormous potential as antimicrobial agents in the food, agriculture, and pharmaceutical industries. Due to the serious threat to human health, the antimicrobial resistance topic has been widely discussed within the scientific community. In this review article, an overview of the recent literature related to the application of citrus-based by-products as antimicrobial agents has been provided. Although it is well known that plant-based by-products exhibit antimicrobial properties under laboratory conditions, more extensive investigations regarding the isolation of specific bioactive compounds, mechanisms of action, in vivo studies, and structure–activity relationship (SAR) analysis are welcomed in the future.

As shown in this review article, citrus by-products can be used as carbon precursors for the preparation of nanoparticles with promising antimicrobial activity, as well as for the production of fluorescent nanoparticles with potential applications as antitumor agents and in cellular imaging. Nowadays, nanoparticles are successfully overcoming the limitations of nonspecific drug delivery and offer multiple benefits in treating human diseases. However, more advanced solutions are needed regarding optimized drug delivery, improvements in their accumulation at the sites of interest, and minimizing unwanted toxicity to ovaerall improve patient outcomes. As plant-based products are generally less toxic in normal cells compared to synthetic compounds, those products show a promising future in medicine, especially with a multidisciplinary collaborative approach of plant science and nanotechnology. With the technological advances, and by applying an interdisciplinary approach, citrus by-products can be a valuable source of compounds with effective antitumor, antioxidant, and protective effects used as health-promoting agents.

## Figures and Tables

**Table 1:** Summary of the literature reporting bioactivities of citrus-based essential oils of the past six years (2018–2023).

Source	Formulation/Chemical Analyte	Bioactivity	Testing Subjects	References
*C. aurantifolia* peel	The essential oil isolated by hydrodistillation using a Clevenger apparatus	Antimicrobial activity	In vitro on multi-drug resistant bacterial isolates	[124]
*C. reticulata* Blanco, *C. aurantifolia* (Christm.) Swingle peel	Essential oils prepared by hydrodistillation	Antimicrobial activity	In vitro against *S. aureus*, including MSSA ^1^, MRSA ^2^, and MDR ^3^ phenotypes, and clinically isolated MRSA and MSSA	[125]
*C. aurantium* “Changshan-huyou” peel	Essential oils isolated by steam distillation	Antimicrobial activity	In vitro against *L. monocytogenes*	[126]
*C. lemon*, *C. aurantifolia*, *C. maxima*, and *C. sinensis* peels	Nano-hexosomal dispersions of citrus essential oils	Antifungal activity	In vitro against phytopathogenic fungi (*R. solani*, *S. rolfsii*, *F. solani*, *F. oxysporum*, *F. semtectium*, *B. cinerea*, and *A. alternata*)	[120]
*C. bergamia* Risso, *C. aurantium* L., *C. sinensis* (L.) Osbeck., *C. deliciosa* Ten., and *C. limon* (L.) Burm. f. peels	Cold-pressed essential oils	Antifungal activity	In vitro against aflatoxin B1 (AFB1)	[127]
*C. bergamia*, *C. sinensis*, *C. limon*, *C. reticulata*, and *C. paradisi* peel	Essential oils obtained by distillation	Antiparasitic activity	In vitro against *Varroa destructor*	[128]
*C. sinensis* peel	The essential oil isolated by hydrodistillation using Clevenger apparatus	Insecticidal activity	In vitro against *Callosobrunchus maculatus* and *Sitophilus zeamais*; studies on the inhibitory effects on acetylcholinesterase (AChE), Na+/K+-ATPase and glutathione-S- transferase (GST) activity	[129]
*C. maxima* peel	Essential oils prepared by hydrodistillation	Insecticidal (larvicidal) activity	In vitro against *Culex tritaeniorhynchus* and *Aedes aegypti* species of mosquitoes	[130]
*C. aurantium* peel	Essential oils prepared by solvent extraction	Antiviral activity	In vitro against influenza A virus H1N1	[131]
*C. clementine* peel	Essential oil prepared by solvent extraction	Antiviral activity	In vitro on Vero-E6 cell lines; SARS-CoV-2 propagated in tested cell line	[132]
Orange, lemon, mandarin, and grapefruit peels ^4^	Commercially purchased essential oils; prepared nanoemulsions	Antioxidant activity	Lipid and fatty acid methyl ester analysis; trout	[133]
*C. reticulata* peel	Essential oil prepared by continuous phase transition extraction	Prevention of hypercholesterolemia and hepatic steatosis	In vivo on male Sprague-Dawley rats on a high-fat diet	[122]
Orange, lemon, mandarin, and grapefruit peels ^4^	Commercially purchased essential oils; prepared nanoemulsions	Suppressive effect on the biogenic amine formation	Trout fillets	[134]
*C. aurantifolia* (Christm.) Swingle peel	Essential oils prepared by steam distillation	Antioxidant capacity and hypolipidemic effect	DPPH^•^, ABTS^•+^ assay; lipid-induced hyperlipidemia in a rat model	[135]
*C. sinensis* (L.) Osbeck	The essential oil isolated by hydrodistillation using a Clevenger apparatus	Antifungal and antitumor activity	Antifungal: *Aspergillus* *carbonarius* and *Aspergillus flavus*/antitumor: Tumor cells (A549, lung adenocarcinoma; MCF-7, breast adenocarcinoma; and HT-144, melanoma) and normal cells (fibroblasts derived from normal human skin, CCD-1059Sk)	[136]
*C. depressa* Hayata pulp	The essential oil isolated by hydrodistillation using a Clevenger apparatus	Stress reduction activity and anti-inflammatory potential	In vivo on nine healthy female panelists (ECG and EEG monitoring); nitric oxide (NO) and interleukin-1β markers	[123]
*C. limon* (L.) Burm f. peel	Commercially purchased essential oil	Anxiolytic and sedative properties	In vivo on Swiss mice model	[137]
*C. reticulata* Blanco peels	The essential oil obtained by supercritical CO_2_ extraction	Mood disorder/relaxing agent	In vivo on adult male Wistar rats; inhalation	[138]
*C. sinensis*, *C. bergamia*, *C. paradisi, C. grandis*, *C. reticulata* Blanco, *C. japonica*, *C. limon*, *C. aurantifolia*, *and immature C. aurantium* L. peels	Essential oils prepared by hydrodistillation	Treatment of dysmenorrhea	In vivo on female Sprague Dawley rats/in vitro on the RL95-2 (human endometrial carcinoma) cells	[139]
*C. limon* peel	Essential oil prepared by steam distillation	The healing effect of traumatic ulcers induced by diabetes	In vivo on diabetic Wistar rats	[140]

^1^ Methicillin-susceptible *S. aureus*; ^2^ methicillin-resistant *S. aureus*; ^3^ multidrug-resistant; ^4^ species not specified.

**Table 4:** Literature reporting the preparation of different (nano)formulations by using citrus by-products as precursors (2018–2023).

Source	Formulation	Application/Bioactivity	Testing Subjects	References
Citrus peel ^1^	Carbon quantum dots conjugated with folic acid	Bioimaging in MCF-7 cell lines, antiradical activity	In vitro on MCF-7 (human breast carcinoma), L929 (mice fibroblasts)	[203]
*C. clementina* peel	Amino acid-functionalized carbon quantum dots	Antiradical activity, bioimaging in MCF-7 cell lines, antitumor activity in pancreatic cancer cell lines	Antiradical activity: DPPH^•^/in vitro on HepG2 (hepatocellular carcinoma), MCF-7 (breast adenocarcinoma, metastatic), HCT-116 (colorectal carcinoma), CFPAC-1 (cystic fibrosis pancreatic adenocarcinoma, metastatic), and HFF-1 (human foreskin fibroblasts)	[204]
Commercial product	Hesperetin cocrystals with piperine	Drug delivery	In vivo bioavailability on Sprague–Dawley rats	[205]
*C. sinensis* peel	Hesperidin nanocrystals	Cosmetics	In vitro on artificial skin	[206]
*C. sinensis* L. Osbeck var. Valencia peel	Hesperidin hexosomal loaded nanodispersion	Antimycobacterial, cytotoxic, and anti-HCov activity	Antimycobacterial: *Mycobacterium (M.) tuberculosis (MTB)*/cytotoxic: against A-549 (human pulmonary adenocarcinoma) cell lines/antiviral: human coronavirus 229E	[207]
*C. reticulata* peel	Hesperidin encapsulated in magnetic casein-CaFe_2_O_4_ nanohybrid carrier	Drug delivery, antitumor activity	In vitro drug release/in vitro on SKOV-3 (human ovarian cancer cell line) and MDA-MB-231 TNBC (human breast cancer cell line)	[56]
*C. sinensis* var. Valencia peel	Gold nanoparticles (AuNPs)	Anti-inflammatory activity	Nitric oxide inhibitory activity, qRT-PCR ^2^, Western blot	[208]
Orange peel ^1^	Hesperidin gold nanoparticles (Hes-AuNPs)	Neuroprotective and antioxidant effects	In vivo on Wistar rats/antioxidant: DPPH^•^ and in vivo studies	[209]
*C. tangerina*, *C. sinensis*, and *C. limon* peel	Silver nanoparticles (AgNPs)	Antimicrobial activity	Antimicrobial: *E. coli* and *S. aureus*	[196]
*C. limon* peel	Silver nanoparticles (AgNPs)	Antimicrobial activity	Antimicrobial: *A. baumannii*, *S. typhimurium*, *E. coli*, *P. aeruginosa*, *S. aureus*, and *P. vulgaris*	[198]
Lemon, tangerine, and orange peel ^1^	Copper oxide nanoparticles (CuONPs)	Antimicrobial activity	Antimicrobial: five strains of Gram-positive (*Enterococcus (E.) faecalis*, *S. aureus*, *L. monocytogenes*, *S. pneumonia* and *Clostridium (C.) perfringens*) and five strains of Gram-negative (*E. coli*, *Moraxella (M.) catarrhalis*, *Salmonella (S.) enterica* subsp. *diarizonae*, *Campylobacter (C.) coli*, and *P. aeruginosa*) bacteria	[210]
*C. hystrix* peel	Encapsulated essential oil into chitosan nanoparticle	Antimicrobial activity	Antimicrobial: *Propionilbacterium (P.) Acnes*	[211]
*C. clementine vesicles*	Exosome-like nano-sized vesicles	Molecular delivery	Proteomic and bioinformatic studies	[212]
*C. sinensis, C. limon, C. paradise, C. aurantium isolated vesicles*	Micro- and nano-sized vesicles	Antitumor activity	In vitro on breast adenocarcinoma (MCF7), human melanoma (A375), lung adenocarcinoma (A549), and human normal skin keratinocyte (HaCat) cells	[213]

^1^ Species not specified; ^2^ real-time quantitative reverse transcription PCR.

## Data Availability

Data sharing not applicable.
